# Crystal structure of phenyl *N*-(3,5-di­methyl­phenyl)carbamate

**DOI:** 10.1107/S2056989017006922

**Published:** 2017-05-12

**Authors:** Y. AaminaNaaz, Subramaniyan Sathiyaraj, Sundararaj Kalaimani, A. Sultan Nasar, A. SubbiahPandi

**Affiliations:** a Department of Physics, Presidency College (Autonomous), Chennai 600 005, India; b Department of Polymer Science, University of Madras, Guindy Campus, Chennai 600 025, India

**Keywords:** crystal structure, carbamate, ester, (di­methyl­phen­yl)carbamate, N—H⋯O hydrogen bonding, C—H⋯π inter­actions, π–π inter­actions

## Abstract

The asymmetric unit of the title compound, C_15_H_15_NO_2_, contains two independent mol­ecules (*A* and *B*). The di­methyl­phenyl ring, the phenyl ring and the central carbamate N—C(=O)—O group are not coplanar. In mol­ecule *A*, the di­methyl­phenyl and phenyl rings are inclined to the carbamate group mean plane by 27.71 (13) and 71.70 (4)°, respectively, and to one another by 84.53 (13)°. The corresponding dihedral angles in mol­ecule *B* are 34.33 (11), 66.32 (13) and 85.48 (12)°, respectively. In the crystal, the *A* and *B* mol­ecules are arranged alternately linked through *N*—*H*⋯*O*(carbon­yl) hydrogen bonds, forming –*A*–*B*–*A*–*B*– chains, which extend along [100]. Within the chains and linking neighbouring chains there are C—H⋯π inter­actions present, forming columns along the *a*-axis direction. The columns are linked by offset π–π stacking inter­actions, forming a three-dimensional network [shortest centroid–centroid distance = 3.606 (1) Å].

## Abstract 2

The asymmetric unit of the title carbamate, contains two independent mol­ecules (*A* and *B*) with similar conformations. In the crystal, they are arranged alternately, forming –*A*–*B*–*A*–*B*– chains linked by *N*—*H*⋯*O*(carbon­yl) hydrogen bonds, which extend along the *a*-axis direction.

## Chemical context   

The The carbamate group is known in biochemistry for its role in biological processes. For example it tunes haemoglobin affinity for O_2_ during physiological respiration (O’Donnell *et al.*, 1979 ▸). Carbamates are widely employed as pharmacological and therapeutic agents (Greig *et al.*, 2005 ▸), to inhibit different enzymes such as acetyl- and butyrylcholinesterases (Darvesh *et al.*, 2008 ▸), cholesterol esterase (Hosie *et al.*, 1987 ▸), elastase (Digenis *et al.*, 1986 ▸), chymotrypsin (Lin *et al.*, 2006 ▸) and fatty acid amide hydro­lase (FAAH) (Kathuria *et al.*, 2003 ▸). In the solid state, the carbamate group acts as both donor and acceptor in hydrogen bonding, favouring the formation of highly stable synthons. Thus, the carbamate group has been proposed as a building block for hydrogen-bonded solids in crystal engineering (Ghosh *et al.*, 2006 ▸). Most carbamate compounds of inter­est are phenyl derivatives, similar to the title compound whose synthesis and crystal structure are reported on herein.
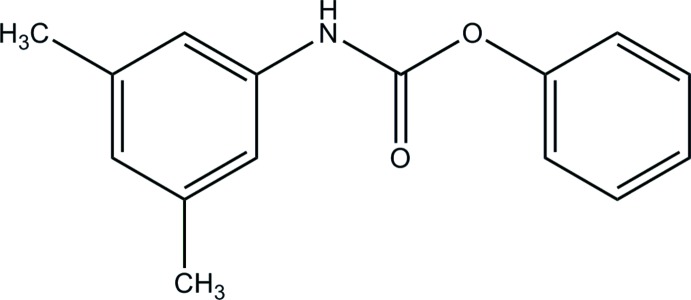


## Structural commentary   

**Figure 1:**
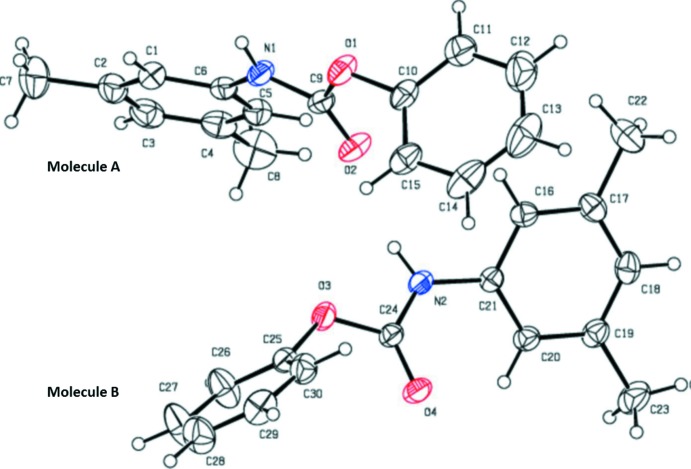
A view of the two independent mol­ecules (*A* and *B*) of the title compound, with the atom labelling. Displacement ellipsoids are drawn at the 30% probability level.

The asymmetric unit of the title compound, Fig. 1 ▸, contains two crystallographically independent mol­ecules (*A* and *B*), with similar conformations. In mol­ecule *A*, the di­methyl­phenyl ring (C1–C6) makes a dihedral angle of 84.53 (13)° with the phenyl ring (C10–C15), and in mol­ecule *B* the di­methyl­phenyl ring (C16–C21) makes a dihedral angle of 85.48 (12)° with the phenyl ring (C25–C30). In mol­ecule *A*, the aryl rings (C1–C6 and C10–C15) are inclined to the the mean plane of the carbamate N1—C9(=O2)—O1 unit by 27.71 (13) and 71.70 (14)°, respectively. In mol­ecule *B*, rings C16–C21 and C25–C39 are inclined to the the mean plane of the carbamate N2—C24(=O24)—O13 unit by 34.33 (11) and 66.32 (13)°, respectively. The C9—N1 and C24—N2 distances are 1.336 (3) and 1.335 (3) Å, respectively, indicating partial double-bond character in the carbamate unit.

## Supra­molecular features   

**Figure 2:**
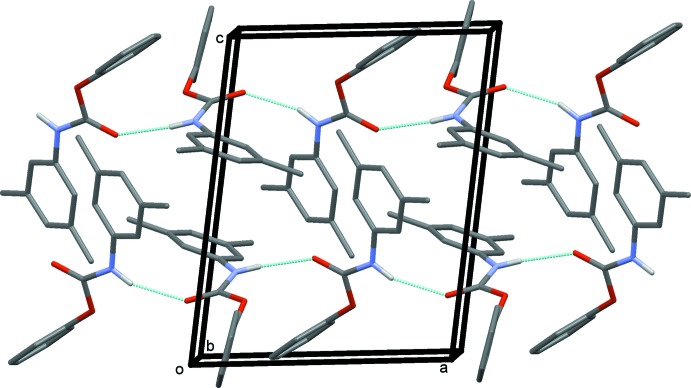
A view along the *b* axis of the crystal packing of the title compound, with the N—H⋯O hydrogen bonds (see Table 1 ▸) shown as dashed lines. For clarity, H atoms not involved in hydrogen bonding have been omitted.

**Figure 3:**
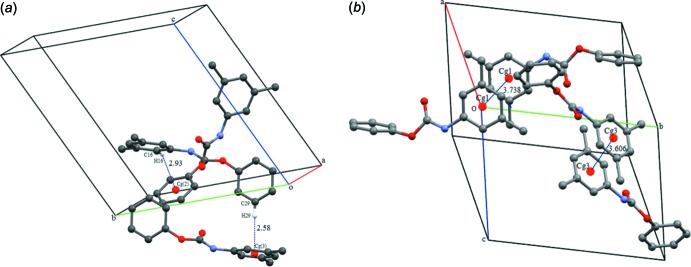
Details of (*a*) the C—H⋯π inter­actions (thin lines; see Table 1 ▸) involving adjacent aromatic rings of the title compound, and (*b*) the offset π–π inter­actions [dotted lines; *Cg*1 and *Cg*3 are the centroids of rings C1–C6 and C16–C21, respectively]. For clarity, H atoms are not involved in these inter­actions have been omitted.

**Figure 4:**
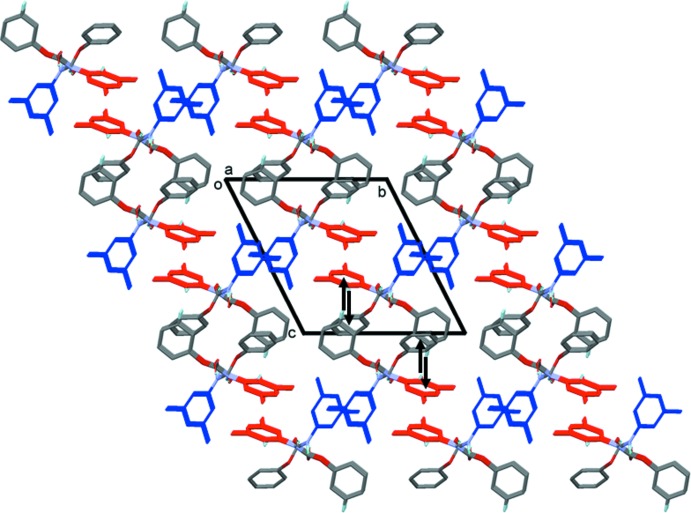
A view along the *a* axis of the crystal packing of the title compound. The hydrogen bonds are shown as dashed lines and examples of the C—H⋯π inter­actions as black arrows (see Table 1 ▸). The rings involved in π–π inter­actions are blue⋯blue (*Cg*1; mol­ecule *A*) and red⋯red (*Cg*3; mol­ecule *B*). For clarity, H atoms are not involved in these inter­actions have been omitted.

In the crystal, N—H⋯O(carbon­yl) hydrogen bonds link the mol­ecules to form –*A*–*B*–*A*–*B*– chains, propagating along the *a*-axis direction (Table 1 ▸ and Fig. 2 ▸). Within the chains and linking neighbouring chains there are C—H⋯π inter­actions, between the H16 and H29 hydrogen atoms of the aromatic and phenyl rings (C10–C15, centroid *Cg*2 and C16–C21, centroid *Cg*3; see Table 1 ▸ and Fig. 3 ▸ *a*). These inter­actions form columns along the *a*-axis direction, which are linked by offset π–π stacking inter­actions (Fig. 3 ▸ *b*), forming a three-dimensional network, as illustrated in Fig. 4 ▸ [*Cg*1⋯*Cg*1^iii^ = 3.738 (2) Å, inter­planar distance = 3.521 (1) Å, slippage = 1.257 Å; *Cg*3⋯*Cg3* ^iv^ = 3.606 (1) Å, inter­planar distance = 3.462 (1) Å, slippage = 1.007 Å; *Cg*1 and *Cg*3 are the centroids of the C1–C6 and C16–C21 rings, respectively; symmetry codes: (iii) −*x* + 3, −*y*, −*z* + 1; (iv) −*x* + 2, −*y* + 1, −*z* + 1].

## Database survey   

A search of the Cambridge Structural Database (Version 5.38, update February 2017; Groom *et al.*, 2016 ▸) for the skeleton phenyl phenyl­carbamate yielded 42 hits. Among these structures there are reports of two *Pna*2_1_ polymorphs of phenyl phenyl­carbamate itself, *viz.* YEHPOQ (Lehr *et al.*, 2001 ▸) and YEHPOQ01 (Shahwar *et al.*, 2009*a* ▸), and those of phenyl (4-methyl­phen­yl)carbamate (YOVHOH; Bao *et al.*, 2009 ▸) and phen­yl(2-methyl­phen­yl)carbamate (YOVLIF; Shahwar *et al.*, 2009*b* ▸). The conformations of all four reported mol­ecules are different. For example, the aromatic rings are inclined to one another by *ca* 25.8° in YEHPOQ, 42.5° in YEHPOQ01, 59.0° in YOVHOH and 39.2° in YOVLIF, compared to 84.5 (1) and 85.5 (1)°, respectively, in mol­ecules *A* and *B* of the title compound.

## Synthesis and crystallization   

To a stirred solution of 1.0 g (5.45 mmol) of 3,5 dimethyl aniline dissolved in 100 ml of dry THF was added a calculated 5% excess of phenyl­chloro­foramate in 50 ml of dry THF. The addition rate was such that it took 1.5 h for complete transfer. After the addition was complete, stirring was continued overnight. Excess THF was removed under vacuum at room temperature. The crude product was extracted with ethyl acetate (3 × 100 ml), and then the organic layer was dried over anhydrous sodium sulfate. Removing the solvent under vacuum at room temperature, yielded a light-yellow product which was dried under vacuum to constant weight. Yellow block-like crystals were obtained by slow evaporation of an ethyl acetate solution at room temperature (yield 99%).

## Refinement   

Crystal data, data collection and structure refinement details are summarized in Table 2 ▸. The N– and C-bound H atoms were positioned geometrically (N—H = 0.86 Å and C—H = 0.93–0.96 Å) and allowed to ride on their parent atoms, with *U*_iso_(H) = 1.5*U*_eq_(C-meth­yl) and 1.2*U*_eq_(N,C) for the H atoms.

## Supplementary Material

Crystal structure: contains datablock(s) global, I. DOI: 10.1107/S2056989017006922/su5370sup1.cif

Structure factors: contains datablock(s) I. DOI: 10.1107/S2056989017006922/su5370Isup2.hkl

Supporting information file. DOI: 10.1107/S2056989017006922/su5370Isup3.cml

CCDC reference: 1548793

Additional supporting information:  crystallographic information; 3D view; checkCIF report

## Figures and Tables

**Table 1:** Hydrogen-bond geometry (Å, °) *Cg*2 and *Cg*3 are the centroids of rings C10–C15 and C16–C21, respectively.

*D*—H⋯*A*	*D*—H	H⋯*A*	*D*⋯*A*	*D*—H⋯*A*
N1—H1⋯O4^i^	0.86	2.14	2.957 (2)	159
N2—H2⋯O2	0.86	2.06	2.896 (2)	164
C16—H16⋯*Cg*2	0.93	2.93	3.659 (2)	136
C29—H29⋯*Cg*3^ii^	0.93	2.59	3.508 (3)	173

Symmetry codes: (i) 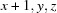; (ii) 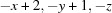.

**Table 2:** Experimental details

Crystal data	
Chemical formula	C_15_H_15_NO_2_
*M*_r_	241.28
Crystal system, space group	Triclinic, *P* 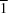
Temperature (K)	293
*a*, *b*, *c* (Å)	9.4257 (4), 12.2054 (5), 13.2067 (6)
α, β, γ (°)	62.979 (3), 82.329 (3), 87.145 (3)
*V* (Å^3^)	1341.29 (10)
*Z*	4
Radiation type	Mo *K*α
μ (mm^−1^)	0.08
Crystal size (mm)	0.20 × 0.18 × 0.17

Data collection	
Diffractometer	Bruker *SMART* APEXII CCD
Absorption correction	Multi-scan (*SADABS*; Bruker, 2008 ▸)
*T*_min_, *T*_max_	0.984, 0.987
No. of measured, independent and observed [*I* > 2σ(*I*)] reflections	31199, 4723, 3376
*R*_int_	0.031
(sin θ/λ)_max_ (Å^−1^)	0.595

Refinement	
*R*[*F* ^2^ > 2σ(*F* ^2^)], *wR*(*F* ^2^), *S*	0.046, 0.143, 1.09
No. of reflections	4723
No. of parameters	325
H-atom treatment	H-atom parameters constrained
Δρ_max_, Δρ_min_ (e Å^−3^)	0.25, −0.20

Computer programs: *APEX2* and *SAINT* (Bruker, 2008 ▸), *SHELXS97* and *SHELXL97* (Sheldrick, 2008 ▸) (Sheldrick, 2008 ▸), *PLATON* (Spek, 2009 ▸) and *Mercury* (Macrae *et al.*, 2008 ▸).

## supplementary crystallographic information

### Crystal data

**Table:**

C_15_H_15_NO_2_	*Z* = 4
*M**_r_* = 241.28	*F*(000) = 512
Triclinic, *P*1	*D*_x_ = 1.195 Mg m^−^^3^
Hall symbol: -P 1	Mo *K*α radiation, λ = 0.71073 Å
*a* = 9.4257 (4) Å	Cell parameters from 3376 reflections
*b* = 12.2054 (5) Å	θ = 1.7–25.0°
*c* = 13.2067 (6) Å	µ = 0.08 mm^−^^1^
α = 62.979 (3)°	*T* = 293 K
β = 82.329 (3)°	Block, yellow
γ = 87.145 (3)°	0.20 × 0.18 × 0.17 mm
*V* = 1341.29 (10) Å^3^	

### Data collection

**Table:**

Bruker SMART APEXII CCD diffractometer	4723 independent reflections
Radiation source: fine-focus sealed tube	3376 reflections with *I* > 2σ(*I*)
Graphite monochromator	*R*_int_ = 0.031
ω and φ scans	θ_max_ = 25.0°, θ_min_ = 1.7°
Absorption correction: multi-scan (*SADABS*; Bruker, 2008)	*h* = −11→11
*T*_min_ = 0.984, *T*_max_ = 0.987	*k* = −14→14
31199 measured reflections	*l* = −15→15

### Refinement

**Table:**

Refinement on *F*^2^	Primary atom site location: structure-invariant direct methods
Least-squares matrix: full	Secondary atom site location: difference Fourier map
*R*[*F*^2^ > 2σ(*F*^2^)] = 0.046	Hydrogen site location: inferred from neighbouring sites
*wR*(*F*^2^) = 0.143	H-atom parameters constrained
*S* = 1.09	*w* = 1/[σ^2^(*F*_o_^2^) + (0.0578*P*)^2^ + 0.5754*P*] where *P* = (*F*_o_^2^ + 2*F*_c_^2^)/3
4723 reflections	(Δ/σ)_max_ < 0.001
325 parameters	Δρ_max_ = 0.25 e Å^−^^3^
0 restraints	Δρ_min_ = −0.20 e Å^−^^3^

### Special details

**Table:**

Geometry. Bond distances, angles etc. have been calculated using the rounded fractional coordinates. All su's are estimated from the variances of the (full) variance-covariance matrix. The cell esds are taken into account in the estimation of distances, angles and torsion angles
Refinement. Refinement of F^2^ against ALL reflections. The weighted R-factor wR and goodness of fit S are based on F^2^, conventional R-factors R are based on F, with F set to zero for negative F^2^. The threshold expression of F^2^ > 2sigma(F^2^) is used only for calculating R-factors(gt) etc. and is not relevant to the choice of reflections for refinement. R-factors based on F^2^ are statistically about twice as large as those based on F, and R- factors based on ALL data will be even larger.

### Fractional atomic coordinates and isotropic or equivalent isotropic displacement parameters (Å^2^)

**Table:**

	*x*	*y*	*z*	*U*_iso_*/*U*_eq_	
O1	1.58017 (15)	0.50462 (14)	0.15809 (14)	0.0562 (5)	
O2	1.41427 (14)	0.39474 (15)	0.30750 (13)	0.0566 (6)	
N1	1.64082 (17)	0.32555 (16)	0.29043 (15)	0.0457 (6)	
C1	1.7338 (2)	0.1239 (2)	0.3932 (2)	0.0532 (8)	
C2	1.7388 (3)	0.0131 (2)	0.4911 (3)	0.0626 (9)	
C3	1.6482 (3)	−0.0046 (2)	0.5884 (2)	0.0642 (9)	
O3	1.16793 (15)	0.29093 (14)	0.18417 (14)	0.0529 (6)	
C4	1.5555 (3)	0.0854 (2)	0.5912 (2)	0.0590 (8)	
O4	0.94345 (14)	0.36607 (14)	0.18663 (13)	0.0491 (5)	
C5	1.5507 (2)	0.1955 (2)	0.4921 (2)	0.0503 (8)	
C6	1.6388 (2)	0.21386 (19)	0.39347 (19)	0.0435 (7)	
C7	1.8376 (4)	−0.0869 (3)	0.4895 (3)	0.0977 (13)	
C8	1.4618 (4)	0.0653 (3)	0.6999 (2)	0.0914 (13)	
C9	1.5337 (2)	0.40501 (19)	0.25803 (18)	0.0420 (7)	
C10	1.4810 (2)	0.5974 (2)	0.10787 (19)	0.0505 (7)	
C11	1.5007 (3)	0.7082 (2)	0.1060 (3)	0.0703 (10)	
C12	1.4096 (4)	0.8039 (3)	0.0507 (3)	0.0948 (13)	
C13	1.3023 (4)	0.7864 (3)	−0.0004 (3)	0.0933 (12)	
C14	1.2842 (3)	0.6744 (3)	0.0022 (2)	0.0795 (12)	
C15	1.3748 (2)	0.5789 (3)	0.0561 (2)	0.0595 (9)	
N2	1.11656 (17)	0.40705 (15)	0.27069 (15)	0.0425 (6)	
C16	1.1256 (2)	0.58070 (18)	0.30825 (17)	0.0422 (7)	
C17	1.0624 (2)	0.66070 (19)	0.34863 (18)	0.0468 (7)	
C18	0.9176 (2)	0.6468 (2)	0.38825 (19)	0.0505 (8)	
C19	0.8359 (2)	0.5547 (2)	0.39031 (18)	0.0467 (7)	
C20	0.9016 (2)	0.47436 (19)	0.35201 (17)	0.0418 (7)	
C21	1.0452 (2)	0.48834 (17)	0.30963 (16)	0.0365 (6)	
C22	1.1512 (3)	0.7594 (2)	0.3502 (3)	0.0725 (10)	
C23	0.6792 (3)	0.5403 (3)	0.4352 (3)	0.0721 (10)	
C24	1.0631 (2)	0.35729 (17)	0.21212 (17)	0.0382 (6)	
C25	1.1406 (2)	0.24132 (19)	0.11142 (18)	0.0417 (7)	
C26	1.1482 (3)	0.1170 (2)	0.1526 (2)	0.0724 (10)	
C27	1.1370 (4)	0.0660 (3)	0.0796 (3)	0.0925 (13)	
C28	1.1152 (3)	0.1379 (3)	−0.0306 (3)	0.0758 (12)	
C29	1.1068 (3)	0.2615 (3)	−0.0702 (2)	0.0630 (9)	
C30	1.1207 (2)	0.3150 (2)	0.0001 (2)	0.0522 (8)	
H1	1.71910	0.34390	0.24420	0.0550*	
H1A	1.79470	0.13780	0.32690	0.0640*	
H3	1.64960	−0.07930	0.65390	0.0770*	
H5	1.48810	0.25680	0.49240	0.0600*	
H7A	1.89210	−0.05870	0.41510	0.1470*	
H7B	1.78230	−0.15830	0.50600	0.1470*	
H7C	1.90150	−0.10750	0.54620	0.1470*	
H8A	1.40440	0.13660	0.68630	0.1370*	
H8B	1.52060	0.05130	0.75850	0.1370*	
H8C	1.40060	−0.00500	0.72410	0.1370*	
H11	1.57400	0.71960	0.14110	0.0840*	
H12	1.42170	0.88010	0.04850	0.1140*	
H13	1.24120	0.85060	−0.03710	0.1120*	
H14	1.21060	0.66280	−0.03260	0.0950*	
H15	1.36370	0.50310	0.05710	0.0710*	
H2	1.20280	0.38790	0.28630	0.0510*	
H16	1.22240	0.58930	0.28020	0.0510*	
H18	0.87400	0.70090	0.41420	0.0610*	
H20	0.84890	0.41050	0.35480	0.0500*	
H22A	1.24840	0.75510	0.31950	0.1090*	
H22B	1.14850	0.74710	0.42760	0.1090*	
H22C	1.11320	0.83870	0.30460	0.1090*	
H23A	0.65130	0.60320	0.45820	0.1080*	
H23B	0.66170	0.46090	0.49980	0.1080*	
H23C	0.62440	0.54770	0.37610	0.1080*	
H26	1.16080	0.06710	0.22880	0.0870*	
H27	1.14440	−0.01860	0.10650	0.1110*	
H28	1.10600	0.10280	−0.07880	0.0910*	
H29	1.09160	0.31090	−0.14580	0.0760*	
H30	1.11650	0.40000	−0.02780	0.0630*	

### Atomic displacement parameters (Å^2^)

**Table:**

	*U*^11^	*U*^22^	*U*^33^	*U*^12^	*U*^13^	*U*^23^
O1	0.0349 (8)	0.0558 (9)	0.0593 (10)	0.0080 (7)	−0.0001 (7)	−0.0123 (8)
O2	0.0302 (8)	0.0715 (11)	0.0554 (10)	0.0058 (7)	−0.0035 (7)	−0.0187 (8)
N1	0.0295 (8)	0.0500 (10)	0.0534 (11)	0.0029 (7)	−0.0028 (8)	−0.0207 (9)
C1	0.0442 (12)	0.0516 (13)	0.0670 (15)	0.0033 (10)	−0.0101 (11)	−0.0291 (12)
C2	0.0626 (15)	0.0462 (14)	0.0798 (19)	0.0059 (11)	−0.0270 (14)	−0.0250 (13)
C3	0.0729 (17)	0.0495 (14)	0.0649 (17)	−0.0067 (13)	−0.0275 (14)	−0.0155 (13)
O3	0.0406 (8)	0.0657 (10)	0.0743 (11)	0.0177 (7)	−0.0184 (7)	−0.0493 (9)
C4	0.0623 (15)	0.0627 (15)	0.0514 (14)	−0.0125 (12)	−0.0131 (12)	−0.0223 (12)
O4	0.0303 (7)	0.0692 (10)	0.0590 (10)	0.0036 (7)	−0.0066 (6)	−0.0388 (8)
C5	0.0461 (12)	0.0536 (13)	0.0551 (14)	−0.0013 (10)	−0.0083 (11)	−0.0272 (12)
C6	0.0359 (10)	0.0447 (12)	0.0534 (13)	−0.0014 (9)	−0.0127 (10)	−0.0231 (11)
C7	0.101 (2)	0.0603 (18)	0.126 (3)	0.0280 (17)	−0.033 (2)	−0.0349 (19)
C8	0.108 (3)	0.099 (2)	0.0551 (17)	−0.0149 (19)	−0.0002 (17)	−0.0254 (17)
C9	0.0308 (11)	0.0498 (12)	0.0473 (12)	0.0006 (9)	−0.0068 (9)	−0.0230 (11)
C10	0.0377 (11)	0.0548 (14)	0.0459 (13)	0.0068 (10)	0.0015 (10)	−0.0138 (11)
C11	0.0558 (15)	0.0610 (16)	0.087 (2)	0.0000 (12)	−0.0054 (14)	−0.0282 (15)
C12	0.084 (2)	0.0554 (18)	0.120 (3)	0.0092 (16)	0.006 (2)	−0.0237 (18)
C13	0.075 (2)	0.086 (2)	0.077 (2)	0.0306 (18)	−0.0041 (17)	−0.0048 (18)
C14	0.0532 (15)	0.123 (3)	0.0531 (16)	0.0237 (17)	−0.0125 (12)	−0.0324 (18)
C15	0.0443 (13)	0.0827 (18)	0.0530 (14)	0.0074 (12)	−0.0024 (11)	−0.0337 (13)
N2	0.0291 (8)	0.0530 (10)	0.0556 (11)	0.0062 (7)	−0.0086 (7)	−0.0332 (9)
C16	0.0387 (11)	0.0465 (12)	0.0404 (12)	−0.0029 (9)	−0.0024 (9)	−0.0192 (10)
C17	0.0582 (13)	0.0401 (11)	0.0411 (12)	−0.0021 (10)	−0.0078 (10)	−0.0169 (10)
C18	0.0579 (14)	0.0536 (13)	0.0463 (13)	0.0118 (11)	−0.0064 (11)	−0.0291 (11)
C19	0.0407 (11)	0.0610 (14)	0.0447 (12)	0.0064 (10)	−0.0052 (9)	−0.0299 (11)
C20	0.0358 (10)	0.0506 (12)	0.0431 (12)	−0.0009 (9)	−0.0025 (9)	−0.0252 (10)
C21	0.0345 (10)	0.0395 (10)	0.0353 (11)	0.0036 (8)	−0.0058 (8)	−0.0168 (9)
C22	0.090 (2)	0.0568 (15)	0.0774 (19)	−0.0152 (14)	−0.0020 (15)	−0.0368 (15)
C23	0.0452 (13)	0.105 (2)	0.085 (2)	0.0088 (13)	0.0023 (13)	−0.0629 (18)
C24	0.0307 (10)	0.0400 (11)	0.0419 (11)	0.0010 (8)	−0.0021 (8)	−0.0176 (9)
C25	0.0350 (10)	0.0473 (12)	0.0492 (13)	0.0049 (9)	−0.0086 (9)	−0.0269 (11)
C26	0.118 (2)	0.0460 (14)	0.0578 (16)	0.0122 (14)	−0.0381 (16)	−0.0215 (12)
C27	0.156 (3)	0.0517 (16)	0.090 (2)	0.0190 (18)	−0.057 (2)	−0.0402 (16)
C28	0.095 (2)	0.081 (2)	0.077 (2)	0.0157 (16)	−0.0331 (16)	−0.0534 (17)
C29	0.0631 (15)	0.0784 (18)	0.0446 (14)	0.0112 (13)	−0.0104 (11)	−0.0253 (13)
C30	0.0514 (13)	0.0453 (12)	0.0529 (14)	0.0058 (10)	−0.0045 (11)	−0.0172 (11)

### Geometric parameters (Å, º)

**Table:**

O1—C9	1.361 (3)	C11—H11	0.9300
O1—C10	1.405 (3)	C12—H12	0.9300
O2—C9	1.205 (2)	C13—H13	0.9300
N1—C6	1.422 (3)	C14—H14	0.9300
N1—C9	1.336 (3)	C15—H15	0.9300
C1—C2	1.387 (4)	N2—H2	0.8600
C1—C6	1.383 (3)	C16—C21	1.381 (3)
N1—H1	0.8600	C16—C17	1.387 (3)
C2—C3	1.379 (4)	C17—C18	1.385 (3)
C2—C7	1.504 (5)	C17—C22	1.510 (4)
O3—C25	1.402 (3)	C18—C19	1.382 (3)
O3—C24	1.365 (3)	C19—C20	1.381 (3)
C3—C4	1.380 (4)	C19—C23	1.504 (4)
C4—C5	1.390 (3)	C20—C21	1.382 (3)
C4—C8	1.505 (4)	C25—C30	1.364 (3)
O4—C24	1.206 (2)	C25—C26	1.361 (4)
C5—C6	1.379 (3)	C26—C27	1.381 (5)
C10—C15	1.366 (3)	C27—C28	1.354 (5)
C10—C11	1.363 (4)	C28—C29	1.356 (5)
C11—C12	1.388 (5)	C29—C30	1.378 (4)
C12—C13	1.363 (5)	C16—H16	0.9300
C13—C14	1.370 (6)	C18—H18	0.9300
C14—C15	1.380 (4)	C20—H20	0.9300
C1—H1A	0.9300	C22—H22A	0.9600
N2—C24	1.335 (3)	C22—H22B	0.9600
N2—C21	1.416 (3)	C22—H22C	0.9600
C3—H3	0.9300	C23—H23A	0.9600
C5—H5	0.9300	C23—H23B	0.9600
C7—H7C	0.9600	C23—H23C	0.9600
C7—H7B	0.9600	C26—H26	0.9300
C7—H7A	0.9600	C27—H27	0.9300
C8—H8A	0.9600	C28—H28	0.9300
C8—H8C	0.9600	C29—H29	0.9300
C8—H8B	0.9600	C30—H30	0.9300
			
C9—O1—C10	117.78 (16)	C14—C15—H15	120.00
C6—N1—C9	126.72 (17)	C10—C15—H15	121.00
C2—C1—C6	120.4 (2)	C21—N2—H2	117.00
C6—N1—H1	117.00	C24—N2—H2	117.00
C9—N1—H1	117.00	C17—C16—C21	120.19 (18)
C3—C2—C7	120.9 (3)	C18—C17—C22	121.3 (2)
C1—C2—C3	118.6 (3)	C16—C17—C18	118.5 (2)
C1—C2—C7	120.5 (3)	C16—C17—C22	120.18 (19)
C2—C3—C4	121.8 (2)	C17—C18—C19	121.9 (2)
C24—O3—C25	118.46 (16)	C20—C19—C23	120.4 (2)
C3—C4—C5	118.9 (2)	C18—C19—C20	118.64 (19)
C3—C4—C8	120.6 (2)	C18—C19—C23	121.0 (2)
C5—C4—C8	120.4 (3)	C19—C20—C21	120.4 (2)
C4—C5—C6	120.0 (2)	C16—C21—C20	120.3 (2)
C1—C6—C5	120.2 (2)	N2—C21—C20	122.0 (2)
N1—C6—C5	122.4 (2)	N2—C21—C16	117.65 (17)
N1—C6—C1	117.29 (19)	O3—C24—O4	123.5 (2)
O1—C9—O2	123.5 (2)	O3—C24—N2	108.53 (17)
O1—C9—N1	109.16 (17)	O4—C24—N2	127.9 (2)
O2—C9—N1	127.3 (2)	O3—C25—C26	117.45 (19)
C11—C10—C15	121.6 (3)	C26—C25—C30	120.9 (2)
O1—C10—C11	117.6 (2)	O3—C25—C30	121.4 (2)
O1—C10—C15	120.6 (3)	C25—C26—C27	119.0 (2)
C10—C11—C12	118.9 (3)	C26—C27—C28	120.7 (4)
C11—C12—C13	120.2 (4)	C27—C28—C29	119.6 (3)
C12—C13—C14	120.2 (3)	C28—C29—C30	120.9 (3)
C13—C14—C15	120.2 (3)	C25—C30—C29	118.9 (3)
C10—C15—C14	119.0 (3)	C17—C16—H16	120.00
C6—C1—H1A	120.00	C21—C16—H16	120.00
C2—C1—H1A	120.00	C17—C18—H18	119.00
C21—N2—C24	126.38 (17)	C19—C18—H18	119.00
C2—C3—H3	119.00	C19—C20—H20	120.00
C4—C3—H3	119.00	C21—C20—H20	120.00
C6—C5—H5	120.00	C17—C22—H22A	109.00
C4—C5—H5	120.00	C17—C22—H22B	109.00
C2—C7—H7C	110.00	C17—C22—H22C	110.00
H7A—C7—H7B	109.00	H22A—C22—H22B	109.00
H7A—C7—H7C	109.00	H22A—C22—H22C	110.00
H7B—C7—H7C	109.00	H22B—C22—H22C	109.00
C2—C7—H7A	109.00	C19—C23—H23A	109.00
C2—C7—H7B	109.00	C19—C23—H23B	110.00
C4—C8—H8A	109.00	C19—C23—H23C	109.00
H8A—C8—H8B	110.00	H23A—C23—H23B	110.00
H8A—C8—H8C	109.00	H23A—C23—H23C	109.00
H8B—C8—H8C	109.00	H23B—C23—H23C	109.00
C4—C8—H8B	109.00	C25—C26—H26	121.00
C4—C8—H8C	109.00	C27—C26—H26	120.00
C10—C11—H11	121.00	C26—C27—H27	120.00
C12—C11—H11	121.00	C28—C27—H27	120.00
C11—C12—H12	120.00	C27—C28—H28	120.00
C13—C12—H12	120.00	C29—C28—H28	120.00
C14—C13—H13	120.00	C28—C29—H29	120.00
C12—C13—H13	120.00	C30—C29—H29	119.00
C13—C14—H14	120.00	C25—C30—H30	120.00
C15—C14—H14	120.00	C29—C30—H30	121.00
			
C10—O1—C9—N1	178.0 (2)	C10—C11—C12—C13	0.2 (5)
C9—O1—C10—C15	−72.6 (3)	C11—C12—C13—C14	−0.3 (5)
C9—O1—C10—C11	111.8 (3)	C12—C13—C14—C15	−0.3 (5)
C10—O1—C9—O2	−3.0 (3)	C13—C14—C15—C10	0.9 (4)
C6—N1—C9—O2	−3.2 (4)	C21—N2—C24—O4	−4.3 (4)
C9—N1—C6—C1	156.0 (2)	C24—N2—C21—C16	−144.0 (2)
C9—N1—C6—C5	−26.5 (4)	C24—N2—C21—C20	38.5 (3)
C6—N1—C9—O1	175.8 (2)	C21—N2—C24—O3	176.05 (18)
C6—C1—C2—C3	−0.6 (4)	C21—C16—C17—C18	−1.0 (3)
C6—C1—C2—C7	177.4 (3)	C21—C16—C17—C22	178.3 (2)
C2—C1—C6—N1	179.3 (2)	C17—C16—C21—N2	−178.04 (18)
C2—C1—C6—C5	1.7 (4)	C17—C16—C21—C20	−0.5 (3)
C7—C2—C3—C4	−179.2 (3)	C22—C17—C18—C19	−178.2 (2)
C1—C2—C3—C4	−1.2 (4)	C16—C17—C18—C19	1.1 (3)
C2—C3—C4—C8	−177.7 (3)	C17—C18—C19—C23	179.2 (2)
C25—O3—C24—O4	7.3 (3)	C17—C18—C19—C20	0.3 (3)
C2—C3—C4—C5	1.9 (4)	C18—C19—C20—C21	−1.8 (3)
C24—O3—C25—C26	−120.5 (2)	C23—C19—C20—C21	179.3 (2)
C25—O3—C24—N2	−173.11 (18)	C19—C20—C21—N2	179.34 (19)
C24—O3—C25—C30	65.6 (3)	C19—C20—C21—C16	1.9 (3)
C3—C4—C5—C6	−0.7 (4)	O3—C25—C26—C27	−173.2 (3)
C8—C4—C5—C6	178.9 (3)	C30—C25—C26—C27	0.7 (4)
C4—C5—C6—C1	−1.1 (3)	O3—C25—C30—C29	174.3 (2)
C4—C5—C6—N1	−178.5 (2)	C26—C25—C30—C29	0.7 (3)
C11—C10—C15—C14	−1.0 (4)	C25—C26—C27—C28	−1.6 (5)
O1—C10—C15—C14	−176.5 (2)	C26—C27—C28—C29	1.2 (5)
O1—C10—C11—C12	176.1 (3)	C27—C28—C29—C30	0.3 (4)
C15—C10—C11—C12	0.5 (4)	C28—C29—C30—C25	−1.2 (4)

### Hydrogen-bond geometry (Å, º)

Cg2 and Cg3 are the centroids of rings C10–C15 and C16–C21, respectively.

**Table:**

*D*—H···*A*	*D*—H	H···*A*	*D*···*A*	*D*—H···*A*
N1—H1···O4^i^	0.86	2.14	2.957 (2)	159
N2—H2···O2	0.86	2.06	2.896 (2)	164
C16—H16···*Cg*2	0.93	2.93	3.659 (2)	136
C29—H29···*Cg*3^ii^	0.93	2.59	3.508 (3)	173

Symmetry codes: (i) *x*+1, *y*, *z*; (ii) −*x*+2, −*y*+1, −*z*.
